# A Qualitative Assessment of a Community Antiretroviral Therapy Group Model in Tete, Mozambique

**DOI:** 10.1371/journal.pone.0091544

**Published:** 2014-03-20

**Authors:** Freya Rasschaert, Barbara Telfer, Faustino Lessitala, Tom Decroo, Daniel Remartinez, Marc Biot, Baltazar Candrinho, Francisco Mbofana, Wim Van Damme

**Affiliations:** 1 Departement of Public Health – Institute of Tropical Medicine, Antwerp Belgium; 2 Médecins Sans Frontières – Tete, Mozambique; 3 Médecins Sans Frontières – Maputo, Mozambique; 4 Médecins Sans Frontières – Brussels, Belgium; 5 Ministery of Health – Tete, Mozambique; 6 Ministery of Health – Maputo, Mozambique; Indiana University and Moi University, United States of America

## Abstract

**Background:**

To improve retention on ART, Médecins Sans Frontières, the Ministry of Health and patients piloted a community-based antiretroviral distribution and adherence monitoring model through Community ART Groups (CAG) in Tete, Mozambique. By December 2012, almost 6000 patients on ART had formed groups of whom 95.7% were retained in care. We conducted a qualitative study to evaluate the relevance, dynamic and impact of the CAG model on patients, their communities and the healthcare system.

**Methods:**

Between October 2011 and May 2012, we conducted 16 focus group discussions and 24 in-depth interviews with the major stakeholders involved in the CAG model. Audio-recorded data were transcribed verbatim and analysed using a grounded theory approach.

**Results:**

Six key themes emerged from the data: 1) Barriers to access HIV care, 2) CAG functioning and actors involved, 3) Benefits for CAG members, 4) Impacts of CAG beyond the group members, 5) Setbacks, and 6) Acceptance and future expectations of the CAG model. The model provides cost and time savings, certainty of ART access and mutual peer support resulting in better adherence to treatment. Through the active role of patients, HIV information could be conveyed to the broader community, leading to an increased uptake of services and positive transformation of the identity of people living with HIV. Potential pitfalls included limited access to CAG for those most vulnerable to defaulting, some inequity to patients in individual ART care and a high dependency on counsellors.

**Conclusion:**

The CAG model resulted in active patient involvement and empowerment, and the creation of a supportive environment improving the ART retention. It also sparked a reorientation of healthcare services towards the community and strengthened community actions. Successful implementation and scalability requires (a) the acceptance of patients as partners in health, (b) adequate resources, and (c) a well-functioning monitoring and management system.

## Introduction

Over the past decade, antiretroviral treatment (ART) coverage has increased significantly in Sub Saharan Africa (SSA) [1]. Decentralisation and task shifting of HIV care and treatment services have contributed to this achievement. Nevertheless, access to and life-long retention on ART remain major challenges [2]–[5]. A systematic review, analysing ART retention in SSA, reported a 76% retention rate at 24 months [6].

In Mozambique, with an adult HIV prevalence of 11.1%, over 1.6 million people are estimated to be HIV positive [1] [7]. By the end of 2012, approximately 283.000 or 48% of the people in need of ART were initiated on treatment [1] [8]–[9]. Of them, only 74% were retained after 12 months on treatment [8]–[9].

The major obstacles and challenges for patients to access and remain on ART in many settings, such as Mozambique, are the large distances to clinics, high transportation costs, long waiting times in clinics, the poor relationship with healthcare workers, cultural beliefs and the preference for traditional medicine [10]–[12].

To overcome some of these patient-reported barriers, the Ministry of Health (MoH) of Mozambique and Médecins Sans Frontières (MSF) piloted a new community-based ‘ART distribution and adherence monitoring model’ through Community ART Groups (CAG). This CAG model was designed to facilitate access to regular drug refills and to reduce the workload in the clinics [13].

To better understand the dynamics, the treatment outcomes and the costs of the CAG model, MSF planned an evaluation from a qualitative, quantitative and costing perspective. The key findings and recommendations derived from the qualitative component of the evaluation, focusing on the relevance, the group dynamic and the impact of the CAG model on individual patients, healthcare services and the broader community, are presented in this manuscript.

## Methods

### Study setting

Mozambique has a population of over 23 million inhabitants and on average 0.3 doctors and 2.1 nurses per 10,000 inhabitants [14]. With more than half of the population living below the poverty line, it remains one of the poorest countries in the world [15]–[16].

Tete province, in the Central region of Mozambique, with 2,137,700 inhabitants has the lowest primary school attendance rate and the lowest female literacy rate in the country (only 25.5% among 15 to 24 year olds) [15] [17]. The adult HIV prevalence is estimated at 7% [18]. Health facilities are very sparse with only one general health facility per 978 km^2^, and one HIV/ART care service per 3874 km^2^.

Since 2002, MSF has supported the MoH with the implementation and scale-up of HIV care and treatment. ART provision started in May 2003 in Tete Provincial Hospital and was decentralised to selected peripheral health facilities in 2006 [18]. Despite this increasing access to ART services, 20% of the ART patients were lost to follow-up and many, especially in rural and remote areas, did not have access to ART [19].

In 2008, to overcome some of the barriers to access and remain on ART, lessons learned from other chronic disease care models were applied to HIV care, engaging people living with HIV (PLHIV) in standardised tasks related to the care of their chronic condition [20]. Mainly the practical peer support to access ART, was expected to result in increased motivation to adhere, better self-management and a reduction of service utilisation [21].

### Description of the CAG model

The CAG model is a community-based ART delivery model. Groups comprise up to six patients on ART. To join a group, patients have to be stable on ART (being at least six months on a first line regimen with a CD4 above 200/mm^3^ and no current opportunistic infections), and live in the same geographical area. Each group elects a group leader among its members, who coordinates the group activities and functions as a spokesperson of the group [13].

**Figure 1:**
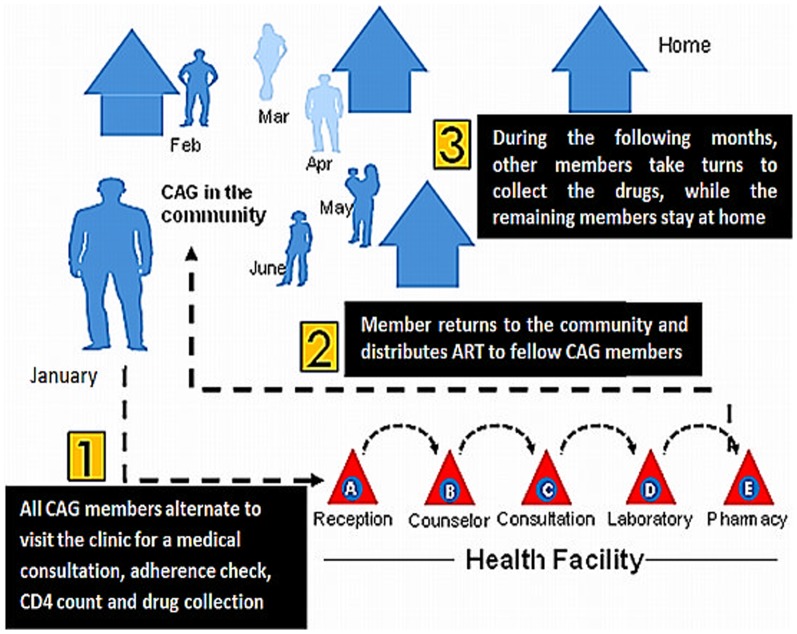
The Community ART Groups model.

Monthly, a group member is appointed to collect the drugs on behalf of the group. In the health facility, he/she receives a medical consultation, reports on the health and the adherence status of his/her fellow group members and collects the drugs for the entire group. On return in the community, (s)he distributes the drugs to the other group members (Figure 1). In the community, the group members meet regularly to check their pill counts, and to counsel each other on adherence issues and daily problems encountered. Healthcare workers, mainly counsellors, sensitise patients to join groups and monitor the group activities. These counsellors, all employed by MSF, are lay people trained and regularly coached by an MSF mobile supervision team. This team monitors and evaluates the quality of care and coordinate the implementation and roll-out of the CAG model.

By December 2012, the CAG model was rolled out in 20 clinics throughout Tete province. In total, over 1,000 groups were formed, representing 5729 adult ART patients: 431 (7.5%) were transferred out, 14 (0.2%) were lost to follow-up and 209 (3.6%) died, with an overall retention rate of 95.7% after a median follow-up time of 19 months in CAG (IQR:10–29). Attrition was 2.2 per 100 person-years, with a mortality rate of 2.1 per 100 person-years and a lost to follow-up rate of 0.1 per 100 person-years. Following this initial success, the MoH incorporated the CAG model into the national HIV strategy in 2012 [22].

### Study design

To assess the relevance, the dynamic and the impact of CAG, we conducted a qualitative study triangulating focus groups discussions (FGD) and in-depth interviews (IDI).

### Sampling methods

The key informants were: (a) patients on ART; (b) MoH nurses; (c) MSF counsellors; (d) health authorities and (e) MSF CAG implementers. The latter group is a core team of MSF workers involved in the initial creation and implementation of the model. These five stakeholder groups were expected to have a good understanding of the CAG model as they are directly and indirectly involved in the CAG model.

Among patients, key informants for the FGDs and IDIs were identified and recruited using a purposive sampling strategies [23]. In the community and the health facilities, respectively counsellors and nurses assisted with the sampling of the patients. The majority of the participants interviewed were patients on ART (79), mostly patients in CAG (68) but also patients who remained in individual care or patients who returned to individual care after leaving CAG. For the health providers (nurses, counsellors and district health authorities), at least one key informant of each health facility where the CAG model was implemented, was recruited. In total, 105 key informants participated in FGD and/or IDI.

### Data collection procedures

Sixteen FGD and 24 IDI were conducted between October 2011 and May 2012 [24]–[25](Table 1). The number of participants in each FGD ranged from three to six participants. To ensure the best possible representation and homogeneity of the participants, we decided to conduct separate FGD with patients - according to their geographical residence (remote, rural and semi-urban areas), counsellors and nurses [26].

**Table 1:** Participants of the focus group discussions and in depth interviews.

Stakeholder groups	Number of IDI	Number of FGD	Number of participants
1. Patients on ART	15	12	79
* In CAG (remote, rural and semi-urban areas)* §	*4*	*12*	*68*
* Returned to individual care*	*4*		*4*
* Remained in individual care*	*7*		*7*
2. MoH nurses*	1	2	10
* Nurses working with counsellors*		1	6
* Nurses working without counsellors*	1	1	4
3. MSF lay counsellors		2	7
4. Health authorities	5		6
* District*	*3*		*3*
* Provincial*	*1*		*1*
* National*	*1*		*2*
5. MSF CAG implementers	3		3
**TOTAL**	**24**	**16**	**105**

CAG – Community ART groups; IDI – In depth interviews; FGD – Focus group discussions.

§ Patients in CAG were divided in three groups according to their geographical residence and the distance to the clinics: (1) remote areas – patients who have to travel long distances to access care with major transport problems, (2) rural areas –patients who can reach healthcare services by foot or bicycle and (3) semi-urban areas – patients who live close to main road with access to public transport.

* To ensure a fluent implementation of the CAG model and monitoring of the groups, MSF appointed counsellors to the large health facilities, taking a major role in the daily management of the CAG activities. Whereas in smaller health facilities, MoH nurses are responsible for all these activities. For the interviews nurses have been divided into two groups: (1) nurses working with counsellors and (2) nurses working without counsellors.

Semi-structured questionnaires for the FGD and IDI with each stakeholder group were elaborated, pre-tested and adapted accordingly (Table 2)[27]. FGD and IDI with nurses, counsellors, health authorities and MSF implementers were conducted in Portuguese while patients were interviewed in their language of choice (Portuguese or a local language). Well-trained moderators assisted by note-takers led the interviews under supervision of an international team of researchers (BT,FR).

**Table 2:** Guiding question topics during interviews and focus group discussions.

Guiding question topics	
**Effectiveness**	How do the CAGs form and function? What is the role of the different CAG members and other stakeholders?
	How are the individual relations and social interactions between the group members?
	What are the inter-variabilities between the different groups according to the health centre/ location they are related to, as well as between the individuals within a same group?
	What is the quality and impact of CAG at clinic, community, and individual levels?
	What is your perception, impression of the CAG model?
**Acceptability**	How do you consider the quality of CAG service delivery: at clinic and community levels?
**Accessibility**	What are the characteristics of people, who do and do not access CAG?
	What are the main barriers to entering CAG? Who should be eligible for CAG?
**Equity**	How is the relationship between patients in CAG and in individual HIV care?
**Relevance**	What are the benefits of CAG?
	How could CAG model better meet the needs of patients, population and health system?
	How do you see the future of the CAG model?

All FGD and IDI were conducted in a quiet and isolated space for an average duration of 60 minutes and were digitally audio-recorded. In addition, note takers took hand-written notes and reported relevant observations.

### Data Analysis

All audio-recorded IDI and FGD were simultaneously translated from local languages into Portuguese and transcribed verbatim using Express Scribe transcription software (NCH Software, Inc. USA) [28]. To ensure the accuracy and completeness of the translations and transcriptions, at least two research team members crosschecked each transcript against the audio-recordings and notes. Corrections to each transcript were made through a group consensus approach.

Inductive qualitative content analysis was used to analyse the data. Three researchers (FL, BT, FR) independently read the transcripts to obtain an overall understanding and overview of the data [29]. Based on these readings a codebook was elaborated and used for the coding of all the transcripts, in NVivo 9 software (QSR International, Doncaster, Vic., Australia) [30]–[31]. All transcripts were divided between the three researchers for coding; this was followed by an extensive coding verification process. Each coded transcript was verified by a second researcher; if needed, a meeting was organised to reach consensus on the code used.

An iterative, cumulative process was applied with regular reflections on the data collected. Based on these preliminary interpretations, triangulating the information retrieved from the different stakeholder groups, questions were adapted or new questions were formulated until a point of data saturation was reached [32]–[33].

Through a process of constant comparison, the data from the different stakeholders was condensed. Key categories were identified, and were then further refined and reduced to the following six themes related to the CAG model: (a) barriers to access HIV care; (b) functioning and actors involved; (c) benefits for the CAG members; (d) impact of CAG beyond the group members; (e) setbacks of the CAG model; and (f) acceptance and future expectations of the CAG model (Table 3).

**Table 3:** Summary of the six themes identified based on the content of the interviews.

Themes	Categories
*1. Barriers to access HIV care*	Resource-limited context with weak healthcare servicesMain barriers to access care: distance, time, cost & stigma
*2. CAG functioning and actors involved in the CAG model*	Eligibility criteria and newly emerged requirements at group levelGroup formation processDifferent roles of stakeholders involved
*3. Benefits for CAG members*	Practical benefitsPsycho-social benefitsSocial control and group rules
*4. Impact of the CAG beyond the group members*	Better health outcomesPatients’ active role in healthcareNew identity of CAG members in group, clinic and communityReduced workload and improved quality of care in clinics
*5. Setbacks of the CAG model*	LimitationsChallengesPotential pitfalls
*6. Acceptance and future expectations of the CAG model*	Thoughts on sustainability of the modelFuture needsFuture adaptation of the CAG model

CAG – Community ART Groups.

### Ethical considerations

This study was approved by the ‘Ethical review boards’ of Mozambique’s MoH (Comite Nacional De Bioetica para a Saúde) and MSF. Local health authorities, healthcare workers and group leaders were informed about the study objectives and procedures of the data collection.

All interviewees were 18 years of age or older. All provided an individual informed consent prior to the data collection. All key informants, not able to read or write, were asked a verbal informed consent, to avoid potential mistrust when signing prior to providing information. This consent procedure was approved by both Ethical review boards. For confidentiality reason, all identifying information was removed during the process.

## Results

### 1. Barriers to access HIV care

Many patients highlighted the difficulties to monthly collect the drugs at the health facilities due to the large distances and related high transport costs to the ART clinics and the time loss in travel and waiting in queues. PLHIV in individual care feel isolated and lack the capacity and support to negotiate their healthcare and to manage their chronic condition on a daily base. They often experience discrimination and social exclusion when monthly attending the clinic. Moreover, many people still strongly believe illnesses are caused by spiritual spells, which can only be managed through cultural beliefs or traditional practices (Table 4.1).

**Table 4:** Quotes of key informants illustrating the major findings.

Themes
**1. Barriers to access HIV care**
*Q1. "…There are many sick people in the villages, of who people in the community think that they are victims of witchcraft without knowing the disease we have…" –* CAG leader during FGD with CAG leaders from semi-urban areas.
*Q2. "…For example if you expose your HIV status, you cannot marry, seduce lovers […], no one will accept you…" –* IDI with CAG member from a rural area
*Q3. "Every month people would wonder…’How am I going to get the drugs? Where will I get the money to pay the transport to go the hospital?’ ” –* CAG member during FDG with CAG members from rural areas.
*Q4. “…in the community there exists some mistrust towards the healthcare workers. Because once you are diagnosed HIV positive in the health facility, there is a feeling that this information will not be kept in the health facility. […] Once the confidence is lost, it is difficult to restore it.”* – IDI with District health authority
*Q5. “Problems of lost to follow up, lack of transport, lack of social support, large distances were the main factors which led to the creation of the CAG model…”* – IDI with MSF CAG implementer
**2. CAG functioning and actors involved in the CAG model**
*Q6. “The majority wants already to join a group after one month on treatment, but we do not accept this. We tell them to complete at least two, three months, then they can join a group.”* – CAG leader during FGD with CAG leaders semi-urban areas
*Q7. "…this month one person goes, another month another person will go, and so on. Normally, when a group member is sick, (s)he has the priority to go to the health facility for a medical consultation. When we are all well, we apply a monthly rotation system.” –* CAG leader during FGD with CAG members from remote areas.
*Q8. “On the day to collect the drugs, the group representative goes directly to the counsellor, who weighs the representative and fills out the clinical fill. […] The counsellor checks for problems in the group. ‘Oh no, there are no problems. You just came to collect the drugs?’ ‘Yes, I came to collect the drugs. Everything is fine.’ So this patient does not need to see the clinician.”* – IDI with counsellor.
**3. Benefits for CAG members**
*Q9. “We as members, we control our colleagues to see if they are adhering or not to the treatment. If someone is not taking it well, we can advise him, saying ‘you have to take the medicines for your health, if you do not take them, you will die.’ Our responsibility is to control each other’s pill counts to see how many pills are left…”* – CAG member during FGD with CAG members from rural areas.
*Q10. “…belonging to a group strengthens people, they become very strong in groups […]. Moreover, being united […] people become mentally stronger compared to the patients in individual care.”* – IDI with CAG leader from a rural area.
*Q11. “With the groups, things have changed because members now do not drink anymore… as drinking is not healthy. […] In the group… we always meet to discuss and advice on our behaviour. If you stop drinking, your health will improve, but if you continue to do what is forbidden, you will always remain ill.”* – IDI with a CAG member from a rural area.
*Q12. “Some people left the groups, because of relationship problems with the group leader.”* – IDI with patient who returned to individual care.
**4. Impact of the CAG beyond the group members**
*Q13. “With the implementation of the CAG model, […] the communication between the patients and the healthcare providers became more intimate. Patients talk openly, they tell everything that hurts, even beyond their disease, they even talk about their social life. The communication between patients and healthcare providers became closer.”* – Nurse during FGD with nurses working without counsellors.
*Q14. “It seems that the discrimination is ending because lately the community seems to lose track of who is receiving treatment as most members look very good. People in the community end up thinking that we are not ill, because before we used to go always to the hospital but now we do not. Some even say we are lying about our HIV status and already cured.” –* CAG member during FGD with CAG members from rural areas
*Q15. “For us healthcare workers, the CAG model reduced the workload, as before we had a queue, a huge queue, from one side to the other, just with patients waiting to collect their drug. Now only the representative for each group appears. It decreased the workload greatly. Even in the pharmacy only one member appears to collect the drugs. So it decreased the workload greatly.”* – IDI with nurse working without counsellor
**5. Setbacks of the CAG model**
*Q16. “As I live next to the clinic, I do not feel the need to join a group.”* – IDI with ART patient who remained in individual care.
*Q17. "…When I am taking ART and I get tuberculosis, I have to stop the ART. I have to take six months of rifampicin. After six months, when I finish, I can start again the ART. Because when you mix ART with other medications or TB treatment it does not work.”* – CAG group member during FGD with CAG members from semi-urban areas.
*Q18. “…When the group member is being assisted, lot of papers need to be revised, the consultation takes usually longer and everyone is waiting. Other patients… they will start making fun of us saying ‘Oh, they are seeing these AIDS people’ You see?” –* IDI with CAG leader from a rural area.
**6. Future expectations of the CAG model**
*Q19. "…in the beginning, I did not… not look very favourably at CAG. I thought it was simply a thing to be introduced. But, the results were coming and are here. I, today, I am almost the defender number one of the CAG model…”* – IDI with District health authority
*Q20. “…training of the group leaders is needed since we are still in the darkness, we do not know many things. Acquiring knowledge is wealth. […] when MSF leaves, we risk to stay without that knowledge. But if we are capacitated, we will remain with the knowledge in our minds.”* – CAG leader during FGD with CAG leaders from semi-urban areas.
*Q21. “I can already see in the future the creation of more groups inspired by the CAG model, for example groups for other chronic diseases,… hypertension, diabetes, cancer, tuberculosis, asthma all inspired by the CAG model… "* – IDI with District health authority

- "*…many people lost their lives because if someone is too weak to go to the health centre, there is no one to help him. Perhaps a patient lives 45km from the health centre. It is far to go alone every month just to collect the drugs, without anyone to help him…*” – IDI with CAG leader from a remote area.

Both patients and health staff considered the healthcare system weak, with a major shortage of health staff and limited infrastructure. The often poorly paid health staff deals with a heavy workload and receives limited training or support.

- "*…the current conditions that are offered by the national healthcare system cannot support the HIV care, […] due to a lack of human resources, lack of infrastructure, and etc. needed to meet the patients’ needs.*" – IDI with National health authority.

These barriers often resulted in many patients mistrusting the healthcare services and opting to abandon or not start at all treatment. Extensive consultations with patient representatives preceded the implementation of this new community-based ART delivery model, to help overcome the patient-reported barriers (Table 4.1).

### 2. CAG functioning and actors involved in the CAG model

Despite clear pre-established medical and geographical eligibility criteria, exceptions to these criteria are often applied such as the timing of acceptance in the CAG. Moreover, several groups have developed their own entry requirements and rules to screen potential new recruits, i.e. the ability to keep secrets, showing a behaviour suitable to the group or being physically well in order to be able to participate in the group activities. In principle, groups can be composed of maximum six members but many groups only count 2 or 3 members, mainly family nuclei (Table 4.2).

- “*First, we have to check the behaviour and attitude of a potential member […] if (s)he is calm, keeps someone’s secret, is not disclosing, […]. It is important not to include someone in the group who cannot keep secrets…*” – CAG leader during FGD with CAG leaders from rural areas.

Generally, groups are formed in the clinics. Counsellors and nurses are very important in brokering the HIV exposure and openness between patients to form groups, establishing trust between patients and moving patient confidentiality from an individual to a group level.

- "*…For us to be able to create a very active group, we depend on the nurses and counsellors. Because when we arrive at the clinic, the nurse asks ‘In what neighbourhood do you live? Do you know this person? Do you know that person?…’ So she fixes a date to return […]. On that date we discover that we are all from the same neighbourhood […]. This makes it possible to meet and form groups.*” – CAG member during FGD with CAG members from semi-urban areas.

Key informants reported on the CAG model responsibilities, shared between MSF counsellors and MoH nurses. Counsellors have a major role in forming, training and monitoring groups at both clinic and community level. They moderate and help solve problems in groups, meet group representatives during the monthly drug collection, exchange information with them and complete group monitoring forms. While, nurses mainly identify potential new group members and refer them to counsellors. They consult and check the CD4 count of individual CAG members every six months.

In several clinics where a counsellor is present, a parallel patient circuit was created, whereby patients in CAG can immediately consult the counsellor to report on the group and receive the drugs without needing a clinical consultation.

### 3. Benefits for the CAG members

Information from CAG key informants show that the CAG model results in some direct practical benefits for patients in groups. The less frequent clinic visits per individual patient reduce the time and cost investment significantly. Also in many groups, members contribute to cover the monthly transport costs of the group representative. Even if sick, occupied, or without money to cover transport costs, group members reported a guaranteed ART supply every month.

- “*There are a lot of advantages being in a group, because in a year… for example I would pay 70, 70 meticais (2.4 USS) on transport cost to go to the hospital. If I have to pay 70 times 12 months, I would spend a lot of money. We had to save money and we worried how to reach the hospital […]. Now we only contribute 10 meticais each month for transport, so in a year we only spend 120 meticais. So with the remaining money, we are able to buy food, maybe vegetables, oil, salt. It is different. Now we only contribute 10 meticais, and it is easy…*” – CAG leader during FGD with CAG leaders from rural areas.

In addition, group members receive and exchange mutual psychosocial support, which is highly valued. They control and advise each other on adherence issues. As a result, patients are found to understand better the importance of the daily drug intake and consequently adhere better to treatment. Being members of a group, patients realise that they are not the only ones living with HIV and needing treatment, which creates a very strong bond and network between the members. The group is often considered as a new family or church, which provides a safe environment to talk freely and exchange information, knowledge and experiences (Table 4.3).

Key informants reported that in many groups, this mutual peer support often evolves into peer pressure and social control to ensure the optimal functioning of the group. Many groups established a code of conduct, which includes fiercely maintaining the group confidentiality, and prohibiting consumption of alcohol or other products (chilli peppers or tobacco) believed to jeopardise the groups’ secrecy or members’ health. Through this code of conduct, group members build a culture of commitment to assure regular drug supply within the group (Table 4.3).

- “*…it is not allowed to disclose the secrets of the group. Similar to the advice one receives when joining the "gule" (traditional secret society), […]. So that is why we trust members […] This trust is based on our friendship and being all in the same situation, needing ART.*" – CAG leader during FGD with CAG leaders from rural areas.

Nevertheless, due to these strict rules or relationship problems in group, some patients preferred to leave the groups and return to individual care (Table 4.3).

### 4. Impact of the CAG model beyond the group members

Overall, the CAG model was perceived as contributing to improved health outcomes. All stakeholders reported better retention on ART with less patients lost to follow-up and/or death in the clinics and communities.

- "*… Before everyone used to suffer alone. If a patient had no money, he would not go to the clinic, that is why there were many deaths. Now the number of deaths decreased because with the groups, we are helping each other.*” – CAG leader during FGD with CAG leaders from rural areas.

Through the CAG model, group members assume several informal healthcare responsibilities in both the clinic and community. For example, they educate and motivate peers, and trace PLHIV lost to follow-up. Some group members help to identify ill people and/or sensitise people in the community to go for HIV testing, which resulted in an increased uptake of healthcare services including HIV testing and care.

- “*…with these groups, there is more dissemination of information. It facilitates the communication. More people are going for counselling and testing and many people are interested to start ART.*” – IDI with District health authority.

The increased involvement of patients in the healthcare services improved significantly the trust and communication between patients in groups and healthcare providers; they often refer to each other as friends or colleagues. In addition, the collective, protective nature of the group allows members the confidence to discuss and negotiate health issues with health authorities or policy makers. (Table 4.4).

- “*Once I went with an ill person to the hospital, the nurse sent him back home and told him to come back tomorrow. I said ‘No! He cannot leave here without treatment…, without any medications’, […] So I went to see the nurse and said: ‘How are you working? This patient need to be seen today and receive medication today!’ […] When there is a problem of patients not being fairly treated by the nurses, the group leader will consult the doctor to present and explain the problem if necessary.*” – CAG leader during FGD with CAG leaders from rural areas.

At the community level, some CAG members have obtained a new identity and a better social status. They are considered active partners in healthcare, and are regularly consulted for health advice, even by HIV negative people. The CAG model also has an impact on the knowledge, attitudes and practices. According to CAG members, the HIV-related stigma and discrimination has reduced, as PLHIV are no longer considered as ill patients. Some key informants even reported an influence of CAG on the conduct of cultural practices, such as widow cleansing, introducing measures to reduce the risk of HIV transmission (Table 4.4).

- “*Eh, specific case of my village. For example myself!… People see me as the first person that any patient or person who is ill in the village should approach. […] Even people with malaria come to my house, they consider me as a nurse, while I am not. I am a CAG leader. I usually explain people the importance to adhere to their treatment and not to consult traditional healers, as it is the only way to salvation.*” – IDI with CAG leader from a rural area.

At the clinic level, the CAG model was found to improve the quality of care. Firstly, the CAG model reduces the workload of the health staff, freeing up more time to attend complicated cases. Secondly, it provides better access to patients’ information regarding pill adherence, wellbeing and treatment outcomes through a direct feedback loop between group members and healthcare workers (Table 4.4).

**Figure 2:**
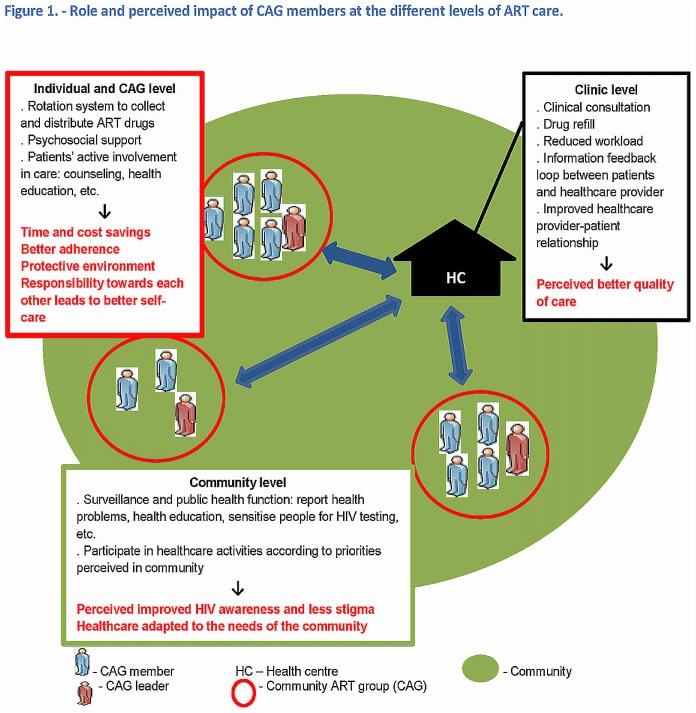
Role and perceived impact of CAG members at the different levels of ART care.

Figure 2 summarises the functioning and the main perceived benefits and impacts of the CAG model at the CAG, clinic and community levels.

### 5. Setbacks of the CAG model

Despite the reported benefits and positive impact of the current CAG approach, different key informants mentioned several limitations and challenges. Some health authorities estimated that the CAG model does not contribute to increased ART access or coverage as all patients are initially started on ART in the clinics, and only join CAG when they are stable on ART. While some patients considered CAG not relevant or acceptable for them. These are mainly patients living close to the clinic or belonging to a higher social class, who do not want to disclose their HIV status out of fear to lose their social status, or who have the means to negotiate their ART supply in another way. Still others simply prefer the individual care delivery system and feel no need to join CAG (Table 4.5).

- "*…For me to return to the group it would not be possible. I am a community leader now.*" – IDI with a patient who returned to individual care.

The most common problems highlighted within the groups were: not keeping group secrets, not participating in the rotation system for drug collection or relationship problems between the group members. Patients who cannot or do not comply with group rules or responsibilities can be requested to leave the group to avoid jeopardising its viability. Moreover, health authorities expressed their fear that the low basic education level of the majority of the CAG members, often reflected in certain patients’ beliefs regarding ART intake, risks to stimulate wrong health messages (Table 4.5).

- “*In the groups, some members will hide! When we inform them that it is their turn to receive the drugs for the group, to collect the drugs, what they do? They run away… they refuse to collect the drugs.*” – CAG group member during FGD with CAG members from semi-urban areas.

Despite the mentioned reduction of discrimination towards HIV patients, HIV-related stigma and the use of traditional healers remain challenges. The lack of privacy at the clinic (for example being exposed when queuing at the pharmacy) is a recurrent problem (Table 4.5).

The CAG model also might create some inequities between patients in CAG and in individual care. For example, in many clinics, CAG members do not need to wait while the majority of patients have to spend hours in queues. It is also evident that in some clinics, CAG is promoted as the best or only ART delivery model. Some key informants even claimed that sometimes patients cannot access ART if they do not join the CAGs.

- “*In my village, it happened that some people refused to be in a group. When they arrived at the hospital to receive drugs they were sent back and told: ‘If you want to receive drugs you have to be in a group’.*”– CAG member during FGD with CAG members from semi-urban areas.

### 6. Acceptance and future expectations of the CAG model

Generally the CAG model is highly accepted and appreciated by all stakeholders. Nevertheless some stakeholders raised doubts regarding its sustainability. The current CAG model is perceived as highly dependent on MSF resources, especially on the counsellors, who are contracted and paid by MSF. In addition, material such as cars and fuel for trainings and supervision visits are required; resources which are not immediately available within the MoH (Table 4.6).

- “*…what I have is a counsellor from MSF. So, the day the contract finishes the groups will disappear…*” – Nurse during FGD with nurses working with counsellors.

According to the CAG members, healthcare workers, health authorities and MSF implementers formal training for health staff, and health and treatment literacy education for patients need to be reinforced in order to optimise the future functioning of the CAG model. The CAG model requires an adequate management and follow up of the groups. This includes regular visits to the community to verify the group’s functioning and treatment outcomes, and identify the needs. Furthermore, to strengthen the CAG model there is a need for improved teamwork, and more co-ownership of the CAG model by MoH staff and patients to manage the daily functioning and monitoring of CAG (Table 4.6).

Key informants of different stakeholder groups made some suggestions regarding future adaptations of the CAG model. To allow more patients to access CAG, the CAG eligibility criteria should be broadened, including patients less than six months on ART or with CD4 <200/mm^3^. Special sub-groups could be formed for TB patients, pregnant women, children or patients on second line ART. However some health authorities did not agree to include the latter two groups as they require some extra clinical attention. Also similar models could be applied for the management of other chronic diseases. In addition, group members could be formally trained in HIV counselling and testing, and management of antiretrovirals and other essential drugs stocks in the community, or could be more involved in income generating projects to finance and sustain the group activities (Table 4.6).

- “*Ehhh…, HIV counselling and testing in the community is already one of the recommendations of the MoH. […] I think it can be done… ehhh, carefully and… and… avoiding excesses and train people so they do this with utmost professionalism possible. It is possible to do this…*” – IDI with District health authority.

## Discussion

The CAG model is an innovative ART delivery model, that applies lessons learned from the management of other chronic diseases and community-based models. Through a patient-centred approach and strong peer support, the model resulted in several direct and indirect benefits and impacts.

At individual level, the main incentives for patients on ART to join groups were the immediate time and cost savings, as before many of them had to travel over 60 km from home to the nearest ART service, and this monthly.

At group level, the mutual psychosocial peer support to deal with daily problems resulted in a better understanding of treatment, and improved adherence and retention on ART. Through their regular meetings group members share, combine and develop their knowledge, experience and personal skills to self-manage their disease. In some groups this support evolved over time into peer pressure and social control, apparently to avoid risky behaviour that could endanger the reliable functioning of the CAG.

At health facility level, the traditional healthcare system approach and the hierarchical patient-provider relation seems to be reversed. Through the newly emergent activities of groups, patients take a more active role in healthcare, resulting in a reduced workload for the healthcare workers and a better quality of care in the health facilities.

And last but not least, at community level, it seems that some groups act as satellites of the healthcare system into previously often inaccessible areas in the community, resulting in a higher uptake of healthcare services. The CAG model is perceived as having strengthened the community action and empowered patients to converse with people in their local communities, health authorities and partner organisations.

Similarly, other community-based ART delivery models in Uganda, Kenya and South Africa found that by providing more responsibility, certainty and control to patients to deal with their chronic condition on a daily basis and access their treatment in a smoother way, lead to improved adherence to treatment and the better health outcomes [34]–[36]. In all these models, peer support and community participation are considered as cornerstones for three main reasons [37]–[39]. First, people are likely to be more motivated to adhere to treatment if they have been involved in the decision making process. Second, the collective resources (time, money, energy, etc.) facilitate access to the healthcare services. Third, many PLHIV have become experts in their chronic condition. Through sharing and combining their knowledge and experiences, they can develop the necessary skills to self-manage their disease [21] [40]–[42].

However, the data analysis revealed some ambiguity in the expression of the identity of people in groups. ART patients in groups on the one hand seem to be proud to be a “member” while on the other hand groups carefully regulate their identity and control who has knowledge of their HIV-infected status. The group often acts as a protective mask or disguise, referred to as Gule or Nyau [43]. This is a secret society among Chewa and Nyanja ethnic groups in parts of Mozambique, Malawi and Zambia, in which the initiated people are not allowed to disclose what they learn during the initiation to out-siders (non-initiated). The initiated individuals who do not keep this secrecy or the non-initiated who try to look into the Gule secrets are severely punished. Maintaining confidentiality of members’ HIV status at the group level is of supreme importance, to protect against stigma and discrimination. Similarly to Anonymous Alcoholics groups in developed countries, there seems to be a major difference between disclosing your status in the ‘protective environment of the group’ and ‘living openly with a disease’.

The extent of disclosure often depends on the individual risk assessment of the potential harms and benefits of disclosure, the stage of readiness and capacity to talk openly outside the group about their own personal situation living with HIV [44]–[48]. Different types of disclosure can be identified, such as disclosure to (a) close confidant, (b) fellow ART patients/peers, (c) healthcare provider, and (d) the broader community. Disclosure to a healthcare provider is necessary and unavoidable for diagnosis, clinical consultation and drug refills. Patients in groups by default have their HIV and ART status disclosed to peer fellow group members and are often encouraged and supported to disclose their status to close family members or confidants. Other studies confirmed that offering a strong social network to patients helps to disclose a patient’s status to the members of the closed network and confidants [44]. Nevertheless, despite the increased community involvement, a CAG does not seem to contribute to the HIV disclosure to the broader community. It, however, offers patients an alternative and somewhat enviable identity as “members”, rather than PLHIV.

Despite the many reported benefits of the CAG model, some potential hazards need to be taken into account when implementing the CAG model. First, the eligibility criteria might exclude the patients most vulnerable to ART drop out from peer support, such as patients newly initiated on ART, poorly adherent to or unstable on ART, favouring the stronger and more compliant patients to enter a group. Moreover, patients with social problems, afraid to disclose their status or consuming considerable amounts of alcohol, could be potentially excluded from joining the groups. Second, the quality of care in CAG needs to be safeguarded as some patients lack a regular medical follow-up because some do not participate in the rotation system to collect drugs or others are only seen by the counsellor, bypassing the nurse to avoid the long queues for a clinical consultation. Future access to viral load, however, could facilitate the patients’ monitoring significantly [49]. Third, in many clinics, CAG members seem to receive preferential attention and privacy over ART patients followed in individual care, potentially resulting in inequities in access to healthcare. Fourth, the CAG model should not be considered as a ‘one fits all approach’. Patients should be offered the choice of which care model fits best to their needs and preferences. Joining a CAG should be entirely voluntarily. Last but not least, a number of necessary conditions needs to be in place for the CAG model to function such as uninterrupted drug supply, access to lab facilities, monitoring and evaluation systems, etc.

These hazards will require ongoing monitoring to ensure the sustainability and quality of care in the long term. However they should be considered with caution, as in reality for many patients, CAG was and is the only way to guarantee regular access to ART due to transport and time costs, distances between communities and clinics, the limited availability of ART services, and the overburdened and understaffed healthcare system.

Until now, MSF has supported the CAG activities, providing additional human, financial and logistical resources. The most crucial resources are (a) the cadre of lay-counsellor, not yet formally recognised by the MoH and (b) the MSF CAG implementing team. In many clinics, counsellors remain the main driver of the CAG model. They play a crucial broker role gathering the patients in groups, and are a direct point of contact for the CAG members in the health facilities. Moreover, they support and monitor groups, train CAG members and intervene where needed, whereas the MSF implementers’ team offers regular support and coaching to implement and monitor the groups. It remains an outstanding question how the MoH will mobilise these additional resources currently not available within the healthcare system and to whom the key responsibilities to form and monitor the groups will be delegated in the future.

Overall there is a considerable push for more community involvement in the healthcare and task shifting to CAG members. Up to date patients in CAG and/or group leaders work on a voluntary basis and are motivated by the direct benefits obtained through the CAG model. Nevertheless, to sustain these activities in the future, sufficient attention should be paid to avoid that the patients’ efforts and time dedicated to the function of the CAG model do exceed the individual benefits (5). Despite the common perception of the CAG model as a useful bridge for many patients to access ART, it should not be considered as an easy solution to fill the gaps of the healthcare system. Rather, it needs to be seen as a complementary ART delivery model in the community [50].

All stakeholder groups called for more training of CAG members, group leaders and health staff working with CAGs. As the CAG model builds on the principles of patients’ self-management, more emphasis should be put on acquiring the essential knowledge and problem-solving skills required to self-manage their disease rather than to overload patients with detailed disease-specific treatment literacy [51]. Consequently, regular monitoring of and support to the CAG group dynamic will be required in the future.

A need emerges thus to identify, define and quantify the essential minimal requirements of the CAG model to function effectively and produce good results; in order to facilitate the development of a feasible approach that the MoH could deliver and scale up independently. In addition, it is recommended to list clear descriptions of roles and functions, and analyse the essential minimal knowledge, skills and support patients on ART in this context need to adequately self-manage their disease.

In the future the CAG dynamic could be further explored, involving patients and community more in the care of HIV, e.g. community-based HIV testing and counselling, detection and management of TB cases or other chronic diseases. Drug refills and monitoring of stable patients on ART could be organised outside the health facilities, in the community, reducing the clinical visits to maximum once yearly.

The main strengths of the study are the large number of FGD and IDI done with a wide range of key stakeholders, the flexibility and dedication of the local research team to produce high quality transcripts for analysis, and the willingness of health authorities and other stakeholders to allow critical analysis. Limitations of the study are that first, all the stages of the study were conducted in the field using locally trained people, with the risk of having to compromise on the state of the art rules of qualitative data collection. Second, all FGD and IDI with patients were performed in local languages and simultaneously translated into Portuguese. Despite the rigorous verification process in place some subtle nuances might have been missed during the verbatim transcribing. Third, we used purposive sampling strategies to identity potential participants among CAG members, using counsellors and nurses to select these candidates. This implies inherently a selection and recall bias as people in favour of the CAG model might be more eager to participate and talk positively about their experiences and opinions related to the CAG model. Fourth, up to date, the CAG model is strongly supported by MSF. Therefore, these positive findings cannot be generalised or extrapolated to other settings, without taking the local context, cultural and resources available into account [37].

It will be important to triangulate these qualitative findings with the other two components of the CAG model evaluation – the quantitative and costing study, to obtain an overall view of the impact of CAG on the patients and the healthcare system, and to stimulate further monitoring and evaluation, and operational research on the CAG-like approach.

## Conclusions

The CAG model, an innovative community-based ART delivery model, seems to result in very high retention outcomes among stable ART patients enlisted in peer support groups in Tete, Mozambique. The time and cost saving benefits were felt to lead to an increased feeling of certainty and control to be able to access ART at all times, making the model accessible, affordable and trustworthy. Moreover, the group dynamic seems to create a protective environment where patients can discuss problems, exchange experiences and support each other. This patient empowerment can potentially contribute to a broader impact in the community, changing patients’ identity from being ignored or excluded to being considered as part of the healthcare system.

However the CAG also requires accountability, with clearly delineated responsibilities at all levels involved, especially a regulatory cadre for the daily management and functioning of the groups and a mobile team to offer regular support and supervision. Potential challenges and future pitfalls such as the exclusion of vulnerable patients groups, the extent of MoH involvement and essential resources required should be monitored and studied closely in order not to jeopardise the future of the CAG model.
